# Correction: Impact of elevated air temperature and drought on pollen characteristics of major agricultural grass species

**DOI:** 10.1371/journal.pone.0261879

**Published:** 2021-12-21

**Authors:** Stephan Jung, Nicole Estrella, Michael W. Pfaffl, Stephan Hartmann, Franziska Ewald, Annette Menzel

## Notice of republication

This article was republished on December 7, 2021, to correct errors in the figure placements. Multiple figures were switched, causing the figures to appear in the incorrect order. Please download this article again to view the correct version. The originally published, uncorrected article and the republished, corrected article are provided here for reference.

## Supporting information

S1 File Originally published, uncorrected article. (PDF)

S2 File Republished, corrected article. (PDF)
