# Supplementary material for: Correction: Impact of elevated air temperature and drought on pollen characteristics of major agricultural grass species
Source: PLoS One. 2021 Dec 21;16(12):e0261879. doi: 10.1371/journal.pone.0261879 (PMC8691635; doi:10.1371/journal.pone.0261879)
Supplement: S1 File — (PDF) [file pone.0261879.s001.pdf]

PLOS ONE

RESEARCH ARTICLE

# Impact of elevated air temperature and drought on pollen characteristics of major agricultural grass species

**Stephan Jung**[1]*, **Nicole Estrella**[1], **Michael W. Pfaffl**[2], **Stephan Hartmann**[3], **Franziska Ewald**[1], **Annette Menzel**[1,4]

**1** Department of Life Science Systems, TUM School of Life Sciences, Technical University of Munich, Freising, Germany, **2** Department of Animal Physiology & Immunology, Technical University of Munich, Freising, Germany, **3** Institute for Plant Production and Plant Breeding, Bayerische Landesanstalt für Landwirtschaft, Freising, Germany, **4** Institute of Advanced Study, Technical University of Munich, Garching, Germany

* sjung@wzw.tum.de

Check for updates

OPEN ACCESS

**Citation:** Jung S, Estrella N, Pfaffl MW, Hartmann S, Ewald F, Menzel A (2021) Impact of elevated air temperature and drought on pollen characteristics of major agricultural grass species. PLoS ONE 16(3): e0248759. https://doi.org/10.1371/journal.pone.0248759

**Editor:** Sergio Rossi, Universite du Quebec a Chicoutimi, CANADA

**Received:** November 10, 2020

**Accepted:** March 4, 2021

**Published:** March 26, 2021

**Copyright:** © 2021 Jung et al. This is an open access article distributed under the terms of the Creative Commons Attribution License, which permits unrestricted use, distribution, and reproduction in any medium, provided the original author and source are credited.

**Data Availability Statement:** All relevant data are within the paper and its Supporting information files.

**Funding:** This work was supported by a MICMoR Fellowship through KIT/IMK-IFU to S. Jung.

**Competing interests:** The authors have declared that no competing interests exist.

## Abstract

Grass pollen allergens are known to be one of the major triggers of hay fever with an increasing number of humans affected by pollen associated health impacts. Climate change characterized by increasing air temperature and more frequent drought periods might affect plant development and pollen characteristics. In this study a one-year (2017) field experiment was conducted in Bavaria, Germany, simulating drought by excluding rain and elevated air temperature by installing a heating system to investigate their effects primarily on the allergenic potential of eight selected cultivars of the two grass species timothy and perennial ryegrass. It could be shown for timothy that especially under drought and heat conditions the allergen content is significantly lower accompanied by a decrease in pollen weight and protein content. In perennial ryegrass the response to drought and heat conditions in terms of allergen content, pollen weight, and protein content was more dependent on the respective cultivar probably due to varying requirements for their growth conditions and tolerance to drought and heat. Results support recommendations which cultivars should be grown preferentially. The optimal choice of grass species and respective cultivars under changing climate conditions should be a major key aspect for the public health sector in the future.

## Introduction

Grass pollen is the major cause of aeroallergen-induced respiratory diseases [1,2]. Besides hay fever, grass pollen can also lead to severe asthmatic reactions in the lower human airways when the pollen structure is decomposed and granules are released [3].

In the last decades the length of the pollen season worldwide has been extended and just comparably, the time period in which pollen allergies occur has been prolonged [4]. These clear changes in the pollen season can be explained by an earlier flowering of plant species

due to global climate warming [5,6] whereas the end of flowering seems to be largely unchanged [7]. Besides longer pollen flight seasons, the pollen production and allergenicity of pollen are influenced by higher atmospheric $CO_2$ concentrations [8] and an increasing number of droughts and elevated temperature [9,10]. The affected areas by such extreme conditions will increase in the next decades as well [11]. A change in the growing conditions and thus competition as well as newly invading species will also lead to a shift in the composition of species [12]. In agricultural grassland systems, extensively planted cultivars such as the early flowering variety Ivana from the species perennial ryegrass (*Lolium perenne* L.) are known to have a low tolerance level against drought due to their e.g. alpine origin with high precipitation quantities and thus their cultivation will be limited in the future. Thus, a wise selection of species and cultivars which are adapted to the altered climate conditions is necessary in order to maintain agricultural yields. It was shown that $C_3$ grasses in general have higher requirements regarding water availability than $C_4$ grasses, thus it is suggested that $C_4$ grasses would be more abundant when water limitation increases [13]. The change in species/cultivar composition and management such as cutting dates on agriculture land and other grassland types might also affect the pollen production and their allergenic potential [14]. Unfavorable conditions during the growing season such as extreme dry periods and heat typically reduce grass growth and might inhibit pollen production, while allergenicity potentially increases due to plant stress [15,16]. A study from Switzerland [17] showed that under the hot and dry conditions in spring and summer 2003 peaks in pollen concentration were already reached in May or beginning of June while the duration of the grass pollen season tended to be shorter than in other years. It was also found that grasses almost stopped growth and pollen production at an early stage by end of June. Accordingly patients allergic to grass pollen had severe symptoms of hay fever in May and June whereas symptoms were reduced towards the end of June.

Temperature and precipitation are the main drivers of plant growth and pollen development [18,19]. It was already shown that herbaceous taxa such as grasses are highly climate sensitive, especially for water availability, compared to other taxa [20]. A long term study [21] on grass pollination at the western Mediterranean coast revealed that elevated minimum temperatures and a rise of precipitation in spring led to higher average pollen concentrations and an earlier ending of pollen season. Other studies reported that an increase in temperature and precipitation intensifies the pollen production of early flowering species, while there is only a small effect on late flowering species [20,22], likely related to differentially impacting cutting dates [14]. The timing of water availability during the growing season also plays an important role for the plant development and affects the number of inflorescences, as it could be shown for tallgrass [23]. Nevertheless the response to the water availability during the growing season was still species-specific [23].

Under extreme growing conditions, e.g. longer drought or warm periods, plants suffer from water stress. It has already been shown that there is a clear link between plant growth and–stress of mesic temperate grasslands [13]. Whether the grass allergens are influenced by plant stress remains unclear, since their basic function inside pollen is unknown for the majority of those allergens. According to other studies the pollen release respectively pollen production is more sensitive to meteorological factors than the allergenicity [24–26]. Nevertheless for ragweed it could be shown that under elevated drought stress the expressed sequence tags (ESTs) encoding allergenic ragweed proteins increased, thus allergen content tended to increase as well [27]. Another study on Arabidopsis and rice revealed that pollen allergens tended to be part of metabolic processes in the pollen cell wall and part of stress responses [28]. These latter two studies indicate that grass pollen allergens might be enhanced under stress conditions as well.

In general, up to 95% of patients allergic to grass pollen possess IgE specific for group 1 allergens and 80% for group 5 allergens, thus these two groups make up the major grass pollen allergens [29–31]. Comparing the analysis of group 1 allergens (Phl p1) and group 5 allergens (Phl p5), the allergen quantification is much easier for group 5 allergens. Phl p1 reaches high homology in various grass species, but the immunodominant positions of the amino acids are different. In consequence the immune response to group 1 allergens might differ between grass species which makes an investigation of Phl p1 quite difficult [32].

The impact of drought and elevated temperature primary on the allergenic potential of different grass species and respective cultivars has up till now only been little examined. In this context it still needs to be clarified whether plant stress induces higher allergenic potentials in grass pollen. To quantify the impact of drought and elevated air temperature, this study conducted a one-year field experiment focusing on the effects of warming and drought on the phenological development, pollen weight, protein content and group 5 allergen content (Phl p5) of selected cultivars from the grass species timothy and perennial ryegrass. We hypothesize that dry conditions and elevated temperatures hamper pollen development and increase the allergen content due to plant stress.

## Material and methods

### Investigated grass species and cultivars

The following grass species and associated cultivars were selected: perennial ryegrass (*Lolium perenne* L.): Honroso, Borsato, Indra and Ivana; timothy (*Phleum pratense* L.): Comer, Lischka, Classic and the timothy grass mixture solely from the provenance Giggenhausen (48.363239˚N, 11.649388˚E); and cocksfoot (*Dactylis glomerata* L.): Musketier, Revolin, Diceros and Lidaglo. The timothy grasses from the provenance Giggenhausen are naturally-occurring grasses which are summarized in the following as one group under the name of their provenance. Due to the use of relatively old seedling material in the Lidaglo cultivar with lower germination rates and slower development in the Revolin and Diceros cultivars, the pollen production of cocksfoot was insufficient, whereby appropriate pollen amounts were produced only on the control plots by the cocksfoot cultivar Musketier. Therefore cocksfoot was excluded from the analysis since no meaningful comparisons were feasible. In the beginning of the survey, the cultivar Classic (timothy) developed more slowly in its early growing stage, i.e. seemed to be undersized and less vital, therefore it was excluded from the phenological, height and photographic recordings. Later on, Classic recovered in all treatments so that this cultivar was included into the pollen sampling and the following analytics.

### Experimental site

The experimental study site (Fig 1) is located at the research station Dürnast (48.404457˚N, 11.690464˚E; 445 m a.s.l.), 50 km north east of Munich, southern Germany. The site is part of the well-maintained Gewächshauslaborzentrum Dürnast of the Technical University of Munich. In total the experimental area covered 80 $m^2$ in which 144 plots (each 65 x 50 cm) were installed, 36 for each of the four treatments (Fig 1 and Table 1). The original loamy soil of previous experiments [33] had been exchanged by more sandy soil material with higher drainage capacity [34]. In the beginning of 2014, this material was classified as loamier sand with a 70% proportion of sand and low phosphorus and potassium content. Before sowing of the grass cultivars in autumn 2016, 5 cm of humus was applied. For each plot 0.54 g pure seeding material from one cultivar was mixed with soya grist and then equally distributed over the respective 65 x 50 cm area. Each treatment comprised respectively four different cultivars each

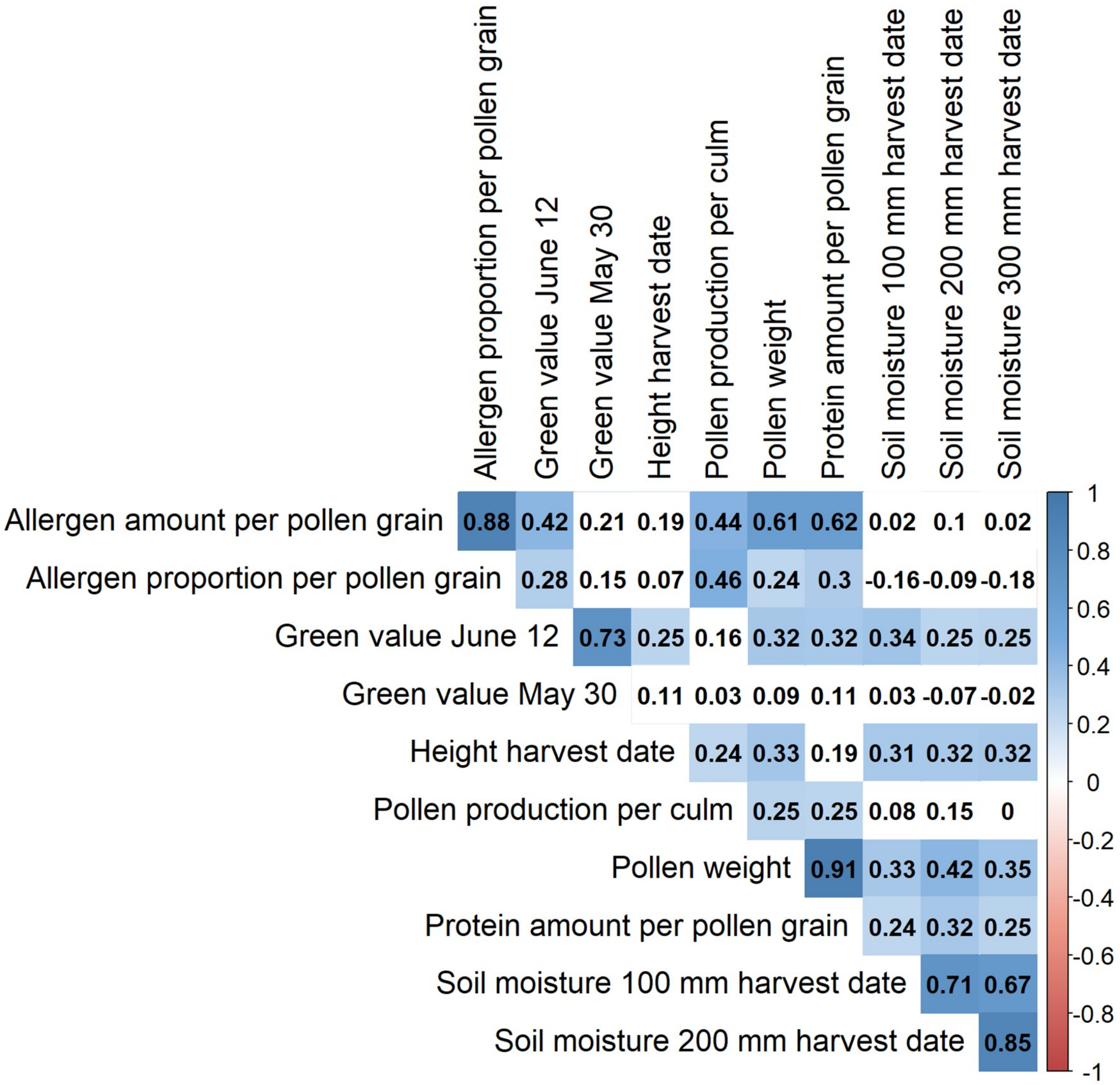


**Fig 1. Experimental setup in Dürnast (48.404457˚N, 11.690464˚E).** Right side: control (red) and warming treatment (green); Left side: drought treatment (blue), warming + drought treatment (yellow area).

https://doi.org/10.1371/journal.pone.0248759.g001

from the three grass species timothy, perennial ryegrass and cocksfoot (4*3 = 12 plots). Within each treatment three repetitions were conducted on a total of 36 plots (see Table 1).

## Treatments

During the investigation period from May 19 until June 23, 2017, the simulation of drought (-stress) and/or elevated air temperature was conducted in three different treatments and one control (Fig 1). Besides the control, the experiment comprised the treatments drought (rain exclusion by a rainout shelter), warming (elevated air temperature) and drought + warming (rain exclusion and elevated air temperature combined).

The elevation of air temperature was achieved by a micro-capillary warm water system (type P.VS30, Beka Heiz- und Kühlmatten GmbH, Berlin, Germany). The capillary mats (5,700 mm length, 630 mm width, capillary tube diameter 4.5 mm, distance between tubes 30 mm) were fixed on a wooden frame in 20 cm height. One single mat covered 9 plots in a row

**Table 1. Experimental setup in Dürnast comprising respectively four different cultivars from the grass species timothy, cocksfoot and perennial ryegrass under three treatments and the control, three repetitions each, cocksfoot was excluded from this study since the pollen production was insufficient.**

| | Control | | | | Warming | | | | Grass species |
|---|---|---|---|---|---|---|---|---|---|
| **Trailer** | Comer | Giggenhausen | Lischka | Classic | Comer | Giggenhausen | Lischka | Classic | Timothy |
| | Musketier | Revolin | Diceros | Lidaglo | Musketier | Revolin | Diceros | Lidaglo | Cocksfoot |
| | Hornroso | Borsato | Indra | Ivana | Hornroso | Borsato | Indra | Ivana | Perennial ryegrass |
| | III. Repetition | | | | | | | | |
| | Comer | Giggenhausen | Lischka | Classic | Comer | Giggenhausen | Lischka | Classic | Timothy |
| | Musketier | Revolin | Diceros | Lidaglo | Musketier | Revolin | Diceros | Lidaglo | Cocksfoot |
| | Hornroso | Borsato | Indra | Ivana | Hornroso | Borsato | Indra | Ivana | Perennial ryegrass |
| | II. Repetition | | | | | | | | |
| | Comer | Giggenhausen | Lischka | Classic | Comer | Giggenhausen | Lischka | Classic | Timothy |
| | Musketier | Revolin | Diceros | Lidaglo | Musketier | Revolin | Diceros | Lidaglo | Cocksfoot |
| | Hornroso | Borsato | Indra | Ivana | Hornroso | Borsato | Indra | Ivana | Perennial ryegrass |
| | I. Repetition | | | | | | | | |
| | **Drought** | | | | **Drought + warming** | | | | |
| **Drought-Shelter** | Comer | Giggenhausen | Lischka | Classic | Comer | Giggenhausen | Lischka | Classic | Timothy |
| | Musketier | Revolin | Diceros | Lidaglo | Musketier | Revolin | Diceros | Lidaglo | Cocksfoot |
| | Hornroso | Borsato | Indra | Ivana | Hornroso | Borsato | Indra | Ivana | Perennial ryegrass |
| | III. Repetition | | | | | | | | |
| | Comer | Giggenhausen | Lischka | Classic | Comer | Giggenhausen | Lischka | Classic | Timothy |
| | Musketier | Revolin | Diceros | Lidaglo | Musketier | Revolin | Diceros | Lidaglo | Cocksfoot |
| | Hornroso | Borsato | Indra | Ivana | Hornroso | Borsato | Indra | Ivana | Perennial ryegrass |
| | II. Repetition | | | | | | | | |
| | Comer | Giggenhausen | Lischka | Classic | Comer | Giggenhausen | Lischka | Classic | Timothy |
| | Musketier | Revolin | Diceros | Lidaglo | Musketier | Revolin | Diceros | Lidaglo | Cocksfoot |
| | Hornroso | Borsato | Indra | Ivana | Hornroso | Borsato | Indra | Ivana | Perennial ryegrass |
| | I. Repetition | | | | | | | | |

https://doi.org/10.1371/journal.pone.0248759.t001

(Fig 1). The warming system was first installed in May 2017, when first grasses reached the height of 20 cm, in order to prevent shading effects as long as possible. In the treatment plots warming and warming + drought, the air temperature at 20 cm height above ground was increased on average by 0.87˚C during the investigation period from May 19 until June 23, 2017.

For the treatments drought and warming + drought, a transparent mobile rainout shelter simulated drought by omitting any precipitation during the investigation period (Fig 1). It was controlled by a rain sensor operating the shelter as soon as the first rain drop hit the sensor. In turn, the shelter reopened again when no further drops hit the sensor.

## Plant development, soil moisture and meteorological data

After sowing in autumn 2016 plants developed well and plots were evenly covered. During the initial growing phase in 2016 and also in spring 2017 all plots were irrigated regularly by a lawn sprinkler for respectively 30 min in the morning and afternoon in order to facilitate plant development. From mid of May till end of June 2017 vegetative and reproductive phenological microstages of each plot were recorded with the expanded BBCH code on a weekly basis following [35]. Observations included all microstages between macrostage 4 (booting), 5 (inflorescence emergence, heading), 6 (flowering, anthesis), 7 (development of fruit), 8 (ripening)

and 9 (senescence). The (micro-) stage was recorded for each plot on a weekly basis. Linear interpolation was used to receive the exact starting dates of flowering (BBCH 61).

The average culm height for each plot was taken in parallel to the phenological recording for each plot. On the respective harvesting dates of the cultivars, inflorescence lengths of the cut culms were measured separately for each plot.

During the investigation period digital images of each plot were taken on May 30 and June 12, 2017. The green value (DN, digital number) of each RGB image (*.jpeg) was extracted and analyzed using the package Fiji [36] which is based on ImageJ [37]. For the interpretation, green values were regarded as proxy for plant vitality.

Between May 19 and June 23, 2017, soil moisture was recorded two to three times per week in depth levels of 100, 200 and 300 mm with a soil moisture sensor (PR2 /6 SDI-12, HH2 Moisture Meter, Delta–T Devices, Cambridge, UK) at 36 spots equally distributed among the treatments where measuring tubes had been embedded in the soil. Due to very low soil water content and clear signs of dehydration of the grasses, all plots were irrigated with a watering can by 1.6 l per plot on June 01, and by 3 l per plot on June 09/14/21, 2017, respectively.

Meteorological data (air temperature, air humidity, and precipitation) in hourly resolution to characterize the growing conditions were obtained from a nearby climate station (Weihenstephan-Dürnast, location 48.4029˚N; 11.7305˚E, distance to field site 385 m) of the German Meteorological Service (DWD). In addition, air temperature and relative air humidity in 20 cm above ground were directly measured by 12 sensors at the site, equally distributed among the treatments.

## Collection of grass pollen and pollen count per blade

In-situ pollen collections are very likely to be influenced by humidity (e.g. caused by rain events), mildew and insects after plants have been covered with collective containers [38]. To overcome these issues, grasses were kept in climate chambers during the actual pollen release. Accordingly, when first flowering (BBCH 61) was observed for a cultivar/treatment, bunches of 10–20 individual plants per plot were harvested and inflorescences were covered with pergamin bags and closed at the bottom. After attaining the full flowering stage (BBCH 65) under fixed conditions in a climate chamber (day/night cycle: 14 hours day at 23˚C, 10 hours night at 15˚C; air humidity 45%), pollen was extracted from the pergamin bags by shaking and subsequent removal of anthers and culms. Additionally, the number of culms per bag was counted and the total pollen weight was determined by an electronic balance (XS204DR, Mettler Toledo GmbH, Gießen, Germany). This method for pollen collection and isolation [39] was consistently applied for all samples. However, it cannot be excluded that single pollen still adhered to the bags or were not released by the anthers. To preserve pollen in the same fresh conditions before analytical testing, they were stored at -20˚C.

## Extraction and determination of protein content

Following Jung et al. [38], pollen grains were mechanically extracted. Total soluble protein content was quantified using BCA test [38]. The reagents (BCA solution and copper sulfate) were ordered from Sigma (B9643; C2284) and for the standard curve Albumin from Serva (11930) was used.

## Grass pollen weight and allergen quantification

Grass pollen weight was measured by dissolving 5 mg pollen grains of each grass cultivar sample immediately before the measurement in 250 μl PBS from which in turn 10μl were counted

(n = 4) using an automated cell counter (TC-10, Bio-Rad Laboratories GmbH, München, Germany) [40]. All samples were counted within one day.

For the quantification of group 5 allergen content a sandwich ELISA (Allergopharma GmbH, Reinbeck/Hamburg, Germany) [41–43] was used, with a sensitivity of 1 ng/ml and precision of ±10% [38,44]. A standard curve was set up by the timothy (*Phleum pretense*) group 5 allergen Phl p5 (Allergopharma GmbH) covering a concentration range of 1 to 1000 ng/ml [38,45]. The epitopes present on the grass pollen allergens Phl p5a and Phl p5b were fixated with the monoclonal antibodies MoAb 1D11 and MoAb B01 (Allergopharma GmbH) and spectrophotometrically visualized with a chromogen present on the biotinylated MoAb B01. Since the group 5 allergens are homologous proteins in grass pollen, the same antibodies could be used for all species of *Poaceae* [46,47].

## Statistical analyses

For the parameters air temperature at 20 cm height, soil moisture, green value, pollen production, pollen weight, protein content and allergen content the Shapiro–Wilk normality test was performed. Since the parameters were not normally distributed (p-value of Shapiro–Wilk test $< 0.05$), non-parametric tests were used. In order to compare more than two groups, the Kruskal–Wallis test was used to check for significant differences (p-value $< 0.05$) between the groups (e.g. temperature at 20 cm height in four treatments). Afterwards, the pairwise Wilcoxon test was chosen to identify significant differences between single pairs of more than two groups. Due to the limited number of samples for the respective treatment and cultivar, differences between treatments were tested on the species level, and not separately for each cultivar. Correlation analysis for non-parametric data was calculated by Spearman's Rank Correlation. P values smaller than 0.05 were considered to be statistically significant. All data were analyzed with R [48] using the packages ggplot2, latticeExtra, tidyr and ggpubr.

# Results

## Growth conditions

According to the data recorded by DWD between April 24 and July 02, 2017, the average air temperature was 15˚C and the average precipitation per day was 2.8 mm (Fig 2). There was a longer dry period between mid-May until end of May with less than 1 mm precipitation in total.

On the plots with warming system air temperature in 20 cm height above ground was on average 0.87˚C higher and relative air humidity 1.34% lower compared to the treatments without warming between May 17 and July 02, 2017 (Fig 2), but these differences between the treatments were not significant (p = 0.325).

Soil moisture in 100, 200 and 300 mm depth was on average 28% lower on the plots where rain was excluded by the rainout shelter ($p<0.001$). During the months May and June soil moisture in 100 mm depth was on average 0.07 $m^3\ m^{-3}$ in the warming treatment, 0.05 $m^3\ m^{-3}$ in the drought treatment, 0.06 $m^3\ m^{-3}$ in the warming + drought treatment and 0.08 $m^3\ m^{-3}$ in the control treatment (Fig 2). The minimum soil moisture of 0.04 $m^3\ m^{-3}$ was reached on June 08 for the drought treatment. Afterwards, around June 14 there was a peak in soil moisture for all treatments due to the irrigation on June 09/14/21, 2017 (see section 2.4).

## Phenological development and height

Among the studied perennial ryegrass cultivars, Ivana started flowering first on May 24 (DOY 144), followed by Indra (DOY 160), Borsato (DOY 164), and Honroso (DOY 167) (Fig 3).

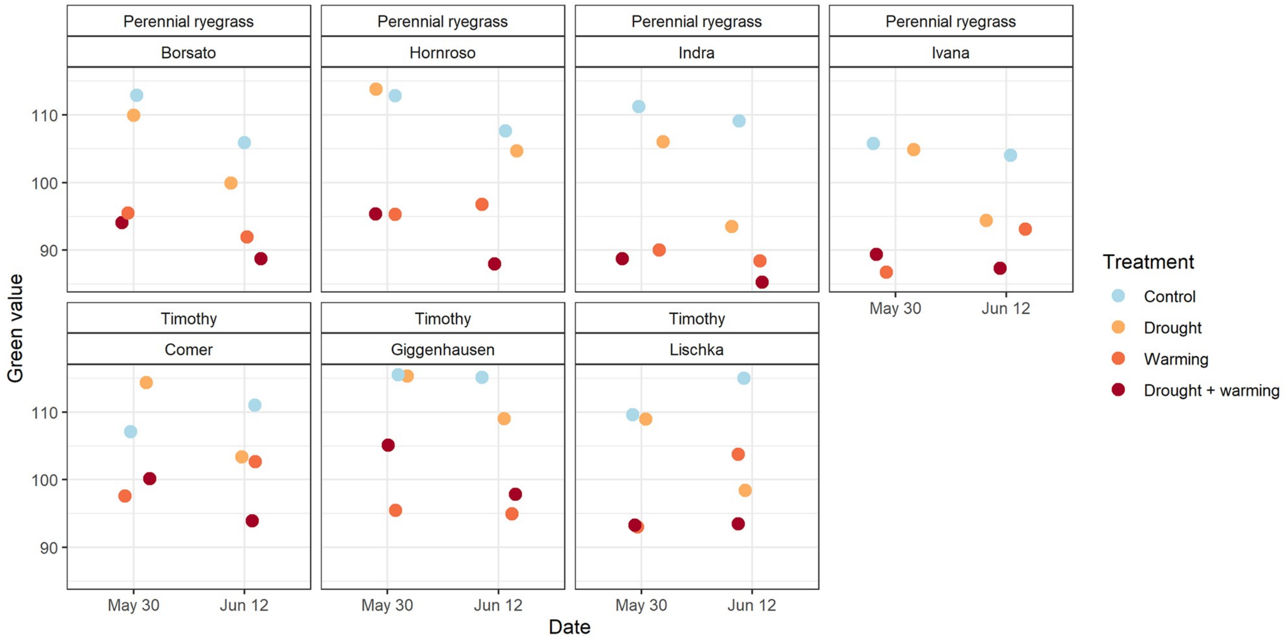


**Fig 2. Meteorological parameters during the investigation period (May 17 to July 02, 2017), top to bottom: Soil moisture [$m^3$ $m^{-3}$] in 100 mm depth (measuring points were only connected for consecutive days, error bars indicate the standard error of 7–9 measurements each), mean daily temperature [˚C] as deviation from the average of all four treatments at 20 cm height (colors represent the treatments, error bars indicate the standard error of 3 measurements each), daily sum of precipitation [mm] and irrigation [mm] (in light blue), daily air temperature [˚C] obtained from the German Meteorological Service (DWD) (in red).**

https://doi.org/10.1371/journal.pone.0248759.g002

Except Ivana, all other cultivars of perennial ryegrass tended to have a slightly faster phenological development in the treatments warming, and drought + warming ($p = 0.47$). In general, the cultivars of timothy started flowering later than perennial ryegrass. Among timothy, Giggenhausen was the first starting to flower on June 13 (DOY 164), second the cultivar Lischka (between DOY 164 and 167), third Comer (DOY 167) and latest Classic (between DOY 167 and 171) (Fig 3). Except for the drought treatment, phenological curve progression was mostly similarly among the other treatments ($p = 0.77$).

Until end of May the average height did not differ between perennial ryegrass (28.5 cm) and timothy (27.6 cm) ($p = 0.74$) (Fig 4). In the period May 17 to June 23, perennial ryegrass grew on average in height by 17.2 cm and timothy by 16.7 cm. Ivana (perennial ryegrass cultivar) had the largest total height (47.8 cm) among all investigated cultivars, whereas Borsato had the lowest total height (33.2 cm). There were no significant differences ($p = 0.75$) among the treatments w.r.t. height growth between May 17 to June 23: the control treatment had on average the strongest increase in height (19.3 cm), followed by the warming (16.6 cm), drought (16.2 cm) and warming + drought (15.2 cm), respectively. Up and downward fluctuations in the height progression can be explained by the (partial) removal of plant individuals at harvesting.

The inflorescence and spikelet length of perennial ryegrass was on average 9.1 cm and for timothy 9.6 cm. There were no significant differences between the treatments. When comparing the treatments, the largest growth in height (37.8 cm) and inflorescence length (13.5 cm) was reached under the control treatment for perennial ryegrass whereas timothy had the largest height (43.5 cm) under the warming treatment and highest inflorescence length under the drought treatment (4.0 cm). There were no significant differences between the treatments for the inflorescence length.

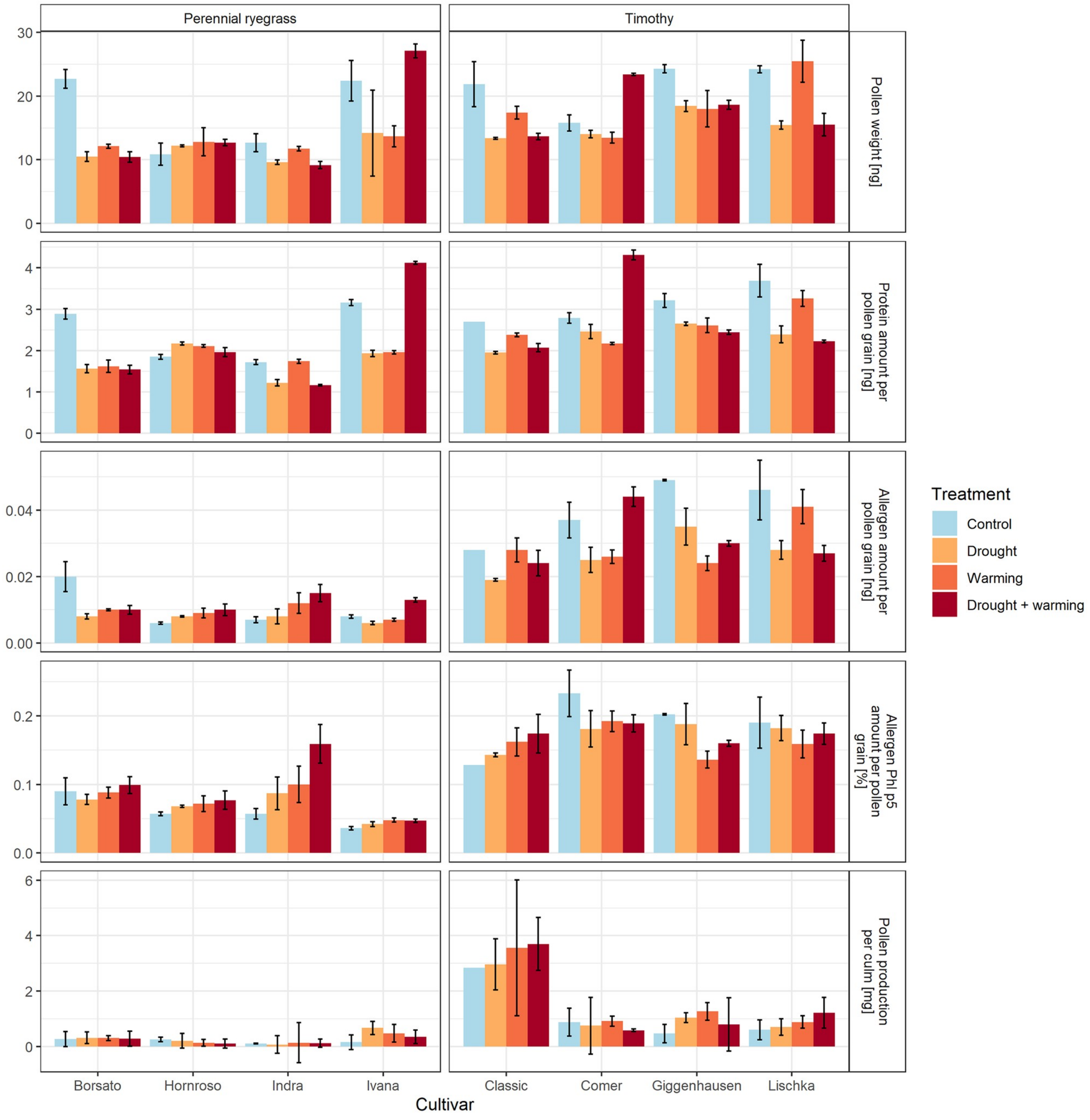


**Fig 3. Phenological development of the cultivars for perennial ryegrass and timothy in spring 2017.** BBCH indicates the (micro-) stages according to BBCH code [35] between May 17 and June 23, 2017. Black lines indicate the beginning of flowering (BBCH 61), parts of the data is missing for the cultivar Classic before its recovery (see Methods 2.1).

https://doi.org/10.1371/journal.pone.0248759.g003

## Pollen analytics

**Weight per grain.** On average the pollen weight per grain was 30% higher in timothy (18.3 ng) compared to perennial ryegrass (14.1 ng, p = <0.01) (Fig 5). Among the observed timothy cultivars, Lischka had the highest pollen weight (20.1 ng) and Classic the lowest (16.6 ng). Among the perennial ryegrass cultivars, Ivana had the highest (19.3 ng) and Indra the

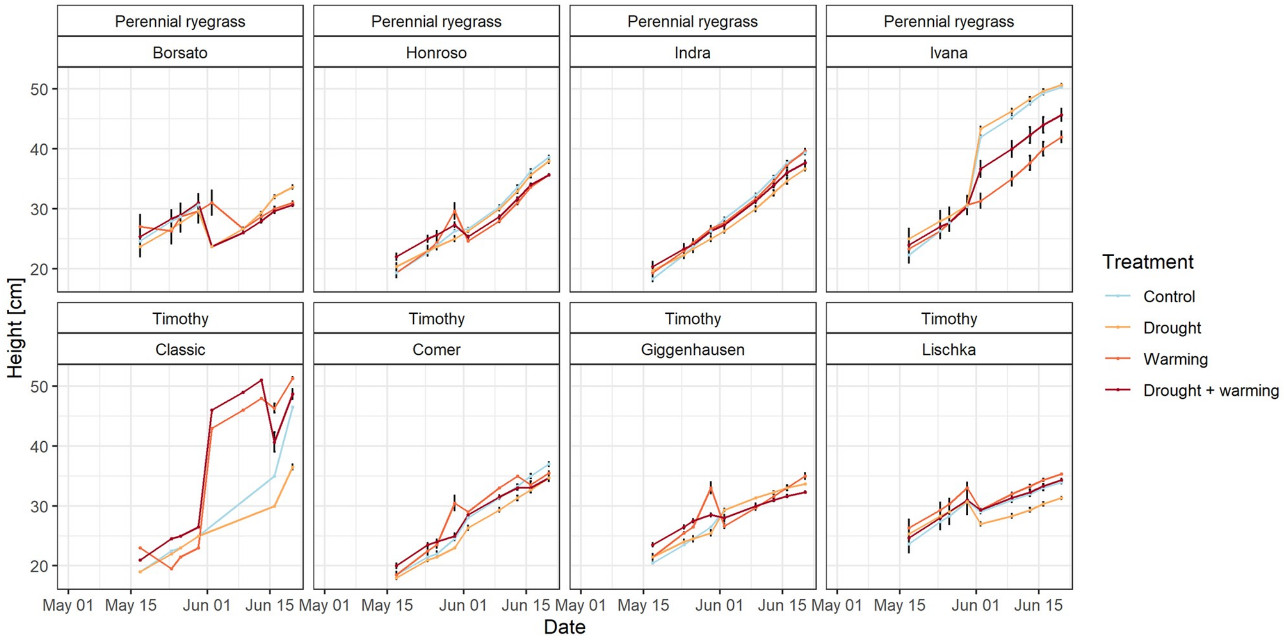


**Fig 4. Height developments of the cultivars of perennial ryegrass and timothy between May 17 and June 23, 2017.** Error bars indicate the standard error of three measurements each.

https://doi.org/10.1371/journal.pone.0248759.g004

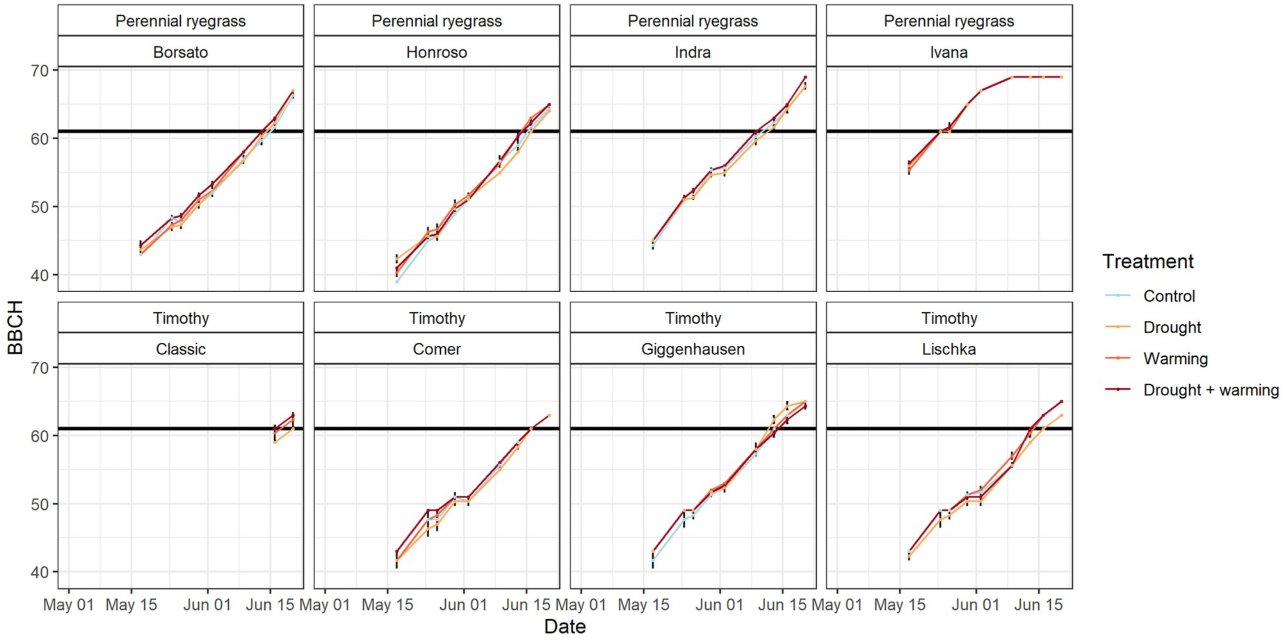


**Fig 5. Pollen characteristics, top to bottom: Pollen weight per pollen grain in [ng], protein amount per pollen grain [ng], allergen amount per pollen grain [ng], allergen Phl p5 amount per pollen grain [%] and pollen production per culm [mg] for perennial ryegrass and timothy.** Colors represent the treatments; error bars indicate the standard error of three repetitions (due to lack of material, Classic control was not repeated except for pollen weight).

https://doi.org/10.1371/journal.pone.0248759.g005

lowest weight per grain (10.8 ng). There were significant differences between the treatments for the perennial ryegrass cultivars ($p<0.05$) and for timothy cultivars ($p<0.01$).

Based on the limited number of samples per treatment and cultivar, statistical tests comparing treatments were carried out for all cultivars of one species combined and not separated by cultivar. For perennial ryegrass significant differences in the weight per pollen grain could particularly be seen between the control (17.2 ng) and the treatments drought (11.6 ng) ($p<0.01$), and drought + warming (12.6 ng) ($p<0.05$). For timothy significant differences were observed between the control (21.5 ng) and the drought treatment (15.3 ng) ($p<0.01$).

**Protein and allergen content.** The average protein amount was 32% higher in timothy (2.7 ng higher per grain) than in perennial ryegrass (2.0 ng per grain, $p = <0.01$) (Fig 5). Regardless of the species, Indra had the lowest protein content (1.5 ng) and Comer the highest (2.9 ng). Protein amount for the timothy cultivars significantly differed between the treatments control (3.1 ng) and drought (2.4 ng) ($p<0.01$). For the perennial ryegrass cultivars there was no significant effect of the treatments.

The absolute allergen content was on average more than three times higher in timothy (0.032 ng) compared to perennial ryegrass (0.010 ng) (Fig 5). The perennial ryegrass cultivar Hornroso had on average the lowest allergen content (0.008 ng) and the timothy cultivar Lischka (0.035) the highest.

The absolute allergen content (ng) was consistently and significantly higher in the control (0.040 ng) in comparison with all treatments for the timothy cultivars (average 0.029) ($p<0.01$). In the case of the perennial ryegrass there was a significant difference between the treatments drought (0.008 ng) and warming + drought (0.012 ng) ($p<0.05$).

On average the allergen proportion (Phl p5) per grain was 57% higher in timothy (0.17% per pollen grain) than in perennial ryegrass (0.08%, $p = <0.001$) (Fig 5). Among the perennial ryegrass cultivars, Ivana had the lowest (0.04%) and Indra the highest content (0.1%). For timothy cultivars, Classic had the lowest (0.15%) and Comer the highest (0.19%).

The allergen proportion (%) of perennial ryegrass cultivars was, with one exception, generally higher in the treatments warming (0.08%), drought (0.10%) and warming + drought (0.07%) than in the control (0.06%) ($p = 0.15$). For timothy cultivars the ranking was opposite, i.e. the allergen proportion for the treatments warming (0.16%), drought (0.17%) and warming + drought (0.17%) was, with one exception, lower than the control (0.19%, $p = 0.15$). However, these differences between the treatments for perennial ryegrass and timothy were not significant ($p = 0.083$ and $p = 0.156$, respectively).

**Pollen per culm.** Highest pollen production was observed for the timothy cultivar Classic (on average 3.26 mg/culm) and lowest for the perennial ryegrass cultivar Indra (on average 0.11 mg/culm) (Fig 5). Timothy grass produced on average much more pollen (1.44 mg/culm) than perennial ryegrass (0.24 mg/culm, $p = <0.001$).

The pollen production per culm for the perennial ryegrass cultivars were on average 25% higher in the treatments control (0.30 mg/culm), drought (0.27 mg /culm) and warming + drought (0.29 mg/culm) than in the treatment warming (0.21 mg/ culm) ($p = 0.51$) (Fig 5). For the timothy cultivars the highest production per culm was found in the warming treatment (1.65 mg/culm), followed by warming + drought (1.57 mg/culm), drought (1.36 mg/culm) and control (1.19 mg /culm), however these treatment differences were not significant ($p = 0.67$).

## Green values

The green value of each plot was determined twice (May 30 and June 12, 2017) by image analysis. For the drought and warming + drought plots, the green value decreased on average by 10% and 5% respectively within these two weeks (Fig 6). In contrast the green values of the

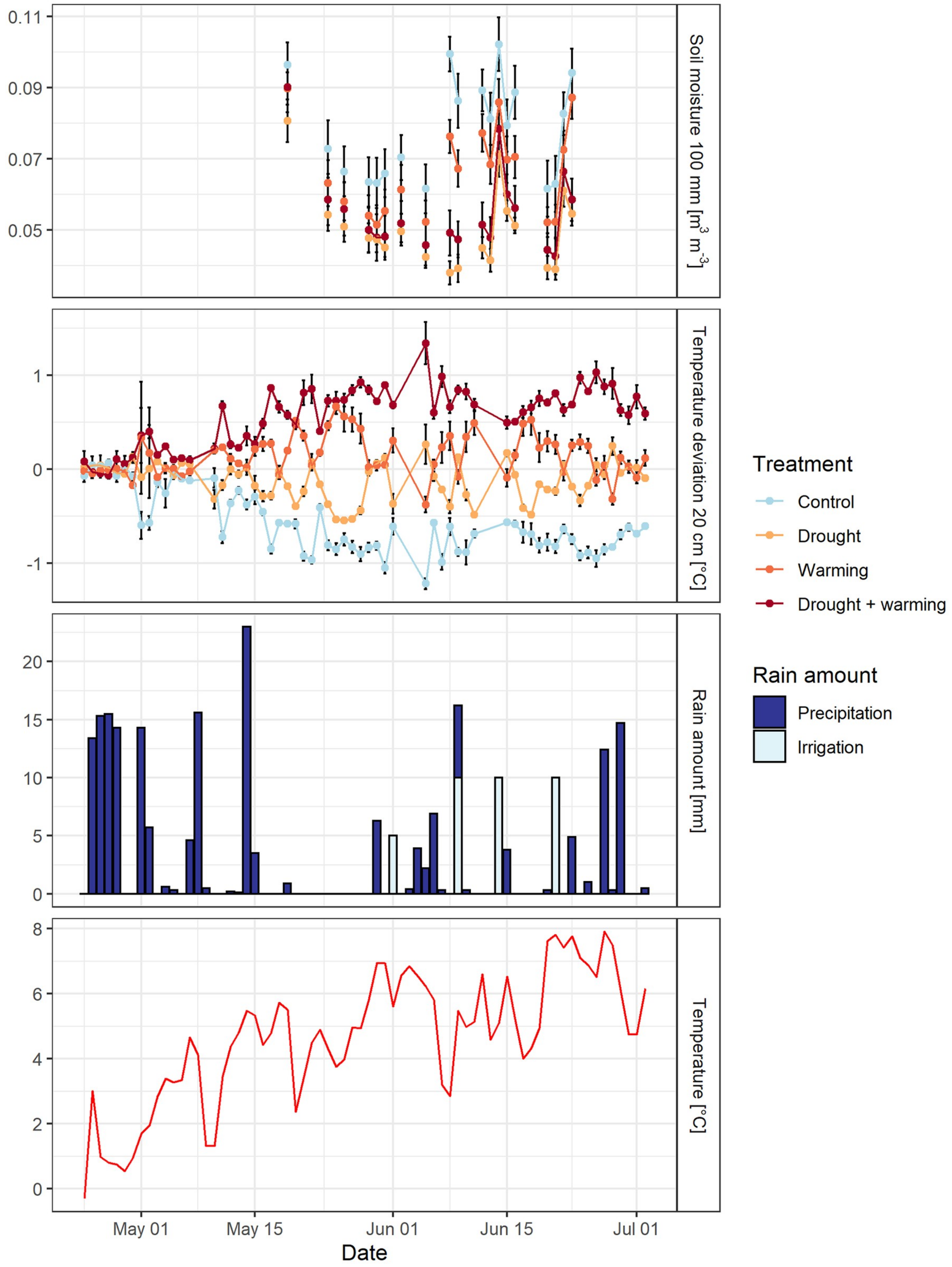


**Fig 6. Green values of the cultivars from (a) perennial ryegrass and (b) timothy on May 30 and June 12 2017, the cultivar Classic (timothy) is missing because it was re-included later into the study again.**

https://doi.org/10.1371/journal.pone.0248759.g006

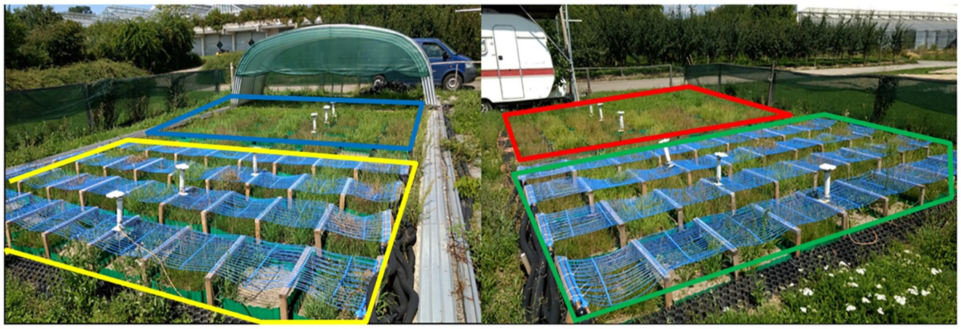

**Fig 7. Spearman correlation including all treatments and the parameters allergen content (ng), allergen percentage (%), green value on May 30 and June 12, height at harvesting date (cm), pollen production per culm (mg), protein content (ng), pollen weight per grain (ng), and soil moisture in 10 cm, 20 cm and 30 cm depth at the harvest date; (level of significance 0.05, significant positive correlations indicated in blue).**

https://doi.org/10.1371/journal.pone.0248759.g007

control and warming plots hardly changed, in case of the timothy cultivars Comer and Lischka the green value even slightly increased (Fig 6). Green values were generally lower (-13%) on the warming treatment plots due to the capillary mats (see Fig 1). The green values for perennial ryegrass significantly decreased between May 30 and June 12 for the control ($p<0.001$), the drought treatment ($p<0.001$) and for the drought + warming treatment ($p<0.05$). For timothy, a significant difference between green values of May 30 and June 12 could only be seen for the drought treatment ($p<0.01$).

## Relationship between pollen characteristics and drought stress

The correlation analysis by Spearman between the soil moisture at all depths and the height of grasses revealed a significant positive correlation ($p<0.05$), same for the green value on June 12 ($p<0.05$) and the pollen protein content ($p<0.05$) (Fig 7). The weight per pollen grain and the protein content were highly significantly and positively correlated ($r = 0.91$, $p = <0.001$), as well as the weight per pollen grain and allergen content ($r = 0.61$, $p<0.001$), and the weight per pollen grain and the height ($r = 0.33$, $p<0.01$) (Fig 7). There was a significant correlation between pollen production per culm and allergen percentage ($r = 0.46$, $p = <0.001$) and respectively allergen content ($r = 0.44$, $p<0.001$). No significant correlation was found between the green values of May 30 and allergen content (ng) ($r = 0.21$, $p = 0.06$) whereas a significant correlation was registered for June 12 ($r = 0.42$, $p<0.001$).

# Discussion

## Comparison with previous studies

Our results demonstrated that climate change related drought and warming clearly influence the development of grass in different ways e.g. by altering the start of flowering, plant development and pollen characteristics. At the same time, the recorded effects of drought and warming were strongly dependent on the grass species/cultivar.

First of all, the results of this study are in close accordance with comparable studies. The collected phenological data are similar to previous observations on plant development and the onset of flowering [38]. Although the grass individuals observed in this study are in the juvenile state, the determined ranges for pollen weight, protein- and allergen content correspond well to those of other studies [38,42]. Solely the error bars for the pollen production are relatively large due to the limited number of samples. Our results reveal significant differences between the grass species perennial ryegrass and timothy, particularly in protein and allergen

contents, which are higher for timothy than for perennial ryegrass, regardless of the treatment. This result for the protein content is consistent with a previous study [49], i.e. the intraspecific variation in pollen production and allergen content is greater in perennial ryegrass than in timothy.

## Grass species and -cultivar dependent response to drought and warming

Elevated temperatures slightly advance the start of flowering of grass species, perennial ryegrass and timothy. This finding supports previous studies, where elevated air temperatures also accelerated the onset of flowering similarly [2,50]. In contrast, the plant development is slightly delayed under the drought treatment with ambient temperatures [51]. The effect of drought becomes evident in the reduction of plant vitality and the markedly change in pollen development.

Pollen weight, protein- and allergen content of the timothy cultivars in drought and warming + drought treatments were in most cases significantly lower than the respective values of the control. Since the probes for pollen weight, protein and allergen content were sampled independently using different analytical methods, a systematic impact of drought on those parameters can be concluded. This systematic effect was found exclusively for timothy, but hardly for the cultivars of perennial ryegrass. The effect itself can be explained by the steadily decreasing plant vitality, which even required additional watering of all plots in mid-June. As most of the perennial ryegrass cultivars started flowering slightly earlier and in the case of Ivana clearly earlier than the timothy cultivars, pollen development was completed before plant vitality was seriously affected. This may explain why systematic effects have been observed for timothy but not for perennial ryegrass. The decrease in plant vitality was also seen in the change of green values between May 30 and June 12 on the plots where rain was excluded. During this period, the green values fell sharply and a slightly slower growth rate was recorded, clearly a sign of water shortage impacting plant development. The comparably high sensitivity of *Poaceae* to drought has already been shown by [20].

The response of the perennial ryegrass cultivars to warming/drought was in comparison to timothy more dependent on the chosen cultivar, but nevertheless a tendency towards higher allergen levels were observed under all three warming/drought treatments. For other pollen characteristics, such as pollen weight, protein and allergen content, no systematic response to the treatments was observed for the perennial ryegrass cultivars. The different reactions of the perennial ryegrass cultivars could be explained by their variable environmental constraints for growth/plant development. In consequence, their individual tolerance levels to drought or elevated temperatures could be very different. This would also explain why drought led to an increase in allergenicity in most of the perennial ryegrass cultivars, whereas a decrease was observed for most of the timothy cultivars. Specifically, the allergen proportion increased under elevated air temperatures/drought for the ryegrass cultivar Indra, Ivana and Hornroso and for the timothy grass cultivar Classic, while a decrease was observed for all other cultivars. To some extent, the influence of elevated air temperature on plant development was certainly overlaid by the interspecific variability between species and cultivars. Regarding pollen production, our results suggest that under elevated air temperatures/drought there is a high dependency on the respective cultivar.

From the climate change perspective our results propose that the allergenic risk due to timothy grass pollen will reduce in the future as the absolute allergen amount decreased under the drought treatment for all four timothy cultivars and under the warming + drought treatment for three cultivars.

The response of perennial ryegrass to drought/warming depends on the cultivar; both increases and decreases of the absolute allergen amount are possible. In comparison to timothy, perennial ryegrass has a much higher number of cultivars with 246 cultivars listed by the Bundessortenamt Germany [52,53], whereby each cultivar is highly specialized to certain environmental conditions. It has to be kept in mind that timothy had up to 5 times higher allergenic content and up to 30 times higher pollen production compared to perennial ryegrass.

## Suggestions for experimental setup

Seeds for our study were obtained from provenances in Bavaria. It cannot be ruled out that specimens of the same species/cultivar but from different provenances show genetic or phenotypic variations and therefore their response to respective treatments might be different. In addition, our study is limited to a one-year field experiment and therefore can only show a portion of the perennial plant's life cycle. It could be possible that the cultivar-/ species-specific patterns shown vary within the life cycle. For example, most of the cocksfoot cultivars cultivated did not flower in the first year regardless of treatment. For validation purposes the experimental setup with the same plants was repeated in 2018. Unfortunately, due to severe technical malfunctions of the climate chambers, results from 2018 were unusable for further analysis.

## Projected changes in future grasslands

Past experimental manipulations of water availability in tallgrass prairie have concluded that water limitations may be important in some years but not in others [23]. Therefore more replications of this experimental approach should be performed in several consecutive years. Due to technical prerequisites and limitations our warming treatment of 0.87˚C in 20 cm height was moderate (comparable to RCP2.6), in future stronger warming scenarios as RCP 4.5 or RCP 8.5 should be considered. The generated drought in this study simulated conditions which can be already found nowadays, also within certain areas in Germany (e.g. $<$30 mm precipitation in May 2017 for Brandenburg and Saxony, Source: German Meteorological Service). Dependent on the soil type and if the generated drought is stronger than in this study ($<$0.04 $m^3$ $m^{-3}$), the permanent wilting point (pF $<$ 4.2) will be reached in certain periods, and grasses in the actual swards might not be able to develop inflorescences and emit pollen any more. Under changing climate with decreases in precipitation, the natural grass species composition will change and adapted cultivars for agricultural production have to be selected. Within the present study we show that depending on the species/cultivars a small increase in temperature and/or drought already has a high impact on the pollen allergenicity. The differences between the species-/ cultivars which revealed in some cases even opposite behavior towards drought/warming, point out the importance of grass species/cultivars selection for people allergic to pollen. In areas where e.g. fodder crops are produced agricultural aspects such as yield, quality parameters and resistance will always be prioritized, but in the development of new cultivars and release of recommendations for adjusted seed mixtures, allergenicity should be at least considered. On the other hand, in areas with extensive use such as landscape lawn or technical grassland the allergenicity of the selected cultivars should be the key factor for decision-making.

# Conclusions

In this study the impact of drought and warming on pollen characteristics such as allergen amount of the two major grass species perennial ryegrass and timothy was examined.

It could be shown that the response to drought and warming is highly dependent on the species and respective cultivars, whereby both increases and decreases of the absolute allergen amount are possible, which can be explained by the cultivar specific requirements for growth conditions and tolerance to drought and heat. For the species timothy the drought and the warming treatment led to significantly lower values for pollen weight, protein content and allergen amount. In comparison, the response to drought and warming of the species perennial ryegrass was highly cultivar-specific. Based on these results, existing knowledge about different grass species and cultivars should be expanded to include the effects of drought and warming on pollen-specific traits such as allergenicity. In the long-run, the outcome of this study can contribute to the development of climate-change adapted seed mixtures which at the same time may not increase the allergenic burden. Under changing climate those aspects have to be well studied within pollen research as they are one of the key factors in public health.

## Supporting information

**S1 File.**
(CSV)

## Acknowledgments

We thank W. Schmid for technical support, and especially Allergopharma GmbH & Co. KG, Reinbeck/Hamburg, Germany for providing the antibodies for the ELISA test.

## Author Contributions

**Conceptualization:** Stephan Jung, Nicole Estrella, Annette Menzel.

**Data curation:** Stephan Jung, Franziska Ewald.

**Formal analysis:** Stephan Jung.

**Funding acquisition:** Annette Menzel.

**Investigation:** Stephan Jung.

**Methodology:** Stephan Jung, Michael W. Pfaffl.

**Project administration:** Nicole Estrella, Annette Menzel.

**Resources:** Michael W. Pfaffl, Stephan Hartmann.

**Supervision:** Nicole Estrella, Annette Menzel.

**Visualization:** Stephan Jung.

**Writing – original draft:** Stephan Jung.

**Writing – review & editing:** Nicole Estrella, Michael W. Pfaffl, Stephan Hartmann, Franziska Ewald, Annette Menzel.

## References

1. Behrendt H B W. Localization, release and bioavailability of pollen allergens: The influence of environmental factors. Current Opinion in Immunology 2001; 13(6):709–15. https://doi.org/10.1016/s0952-7915(01)00283-7 PMID: 11677094
2. D'Amato G, Spieksma FT, Liccardi G, Jäger S, Russo M, Kontou-Fili K, et al. Pollen-related allergy in Europe. Allergy 1998; 53(6):567–78. https://doi.org/10.1111/j.1398-9995.1998.tb03932.x PMID: 9689338

3. Suphioglu C, Singh MB, Taylor P, Knox RB, Bellomo R, Holmes P, et al. Mechanism of grass-pollen-induced asthma. The Lancet 1992; 339(8793):569–72. https://doi.org/10.1016/0140-6736(92)90864-y PMID: 1347092

4. Anderegg WRL, Abatzoglou JT, Anderegg LDL, Bielory L, Kinney PL, Ziska L. Anthropogenic climate change is worsening North American pollen seasons. Proc Natl Acad Sci U S A. 2021; 118. https://doi.org/10.1073/pnas.2013284118 PMID: 33558232

5. Menzel A, Sparks TH, Estrella N, Koch E, Aasa A, Ahas R et al. European phenological response to climate change matches the warming pattern. Global Change Biol 2006; 12(10):1969–76.

6. Emberlin J. The effects of patterns in climate and pollen abundance on allergy. Allergy 1994; 49 (s18):15–20. https://doi.org/10.1111/j.1398-9995.1994.tb04233.x PMID: 8053536

7. Menzel A, Ghasemifard H, Yuan Y and Estrella N. A first pre-season pollen transport climatology to Bavaria, Germany. Front. Allergy: 2:627863. https://doi.org/10.3389/falgy.2021.627863

8. Ziello C, Sparks TH, Estrella N, Belmonte J, Bergmann KC, Bucher E, et al. Changes to airborne pollen counts across Europe. PLoS One 2012; 7(4):e34076. https://doi.org/10.1371/journal.pone.0034076 PMID: 22514618

9. D'Amato G, Cecchi L, Bonini S, Nunes C, Annesi-Maesano I, Behrendt H et al. Allergenic pollen and pollen allergy in Europe. Allergy 2007; 62(9):976–90. https://doi.org/10.1111/j.1398-9995.2007.01393.x PMID: 17521313

10. Beggs PJ. Impacts of climate change on aeroallergens: past and future. Clinical and Experimental Allergy 2004; 34(10):1507–13. https://doi.org/10.1111/j.1365-2222.2004.02061.x PMID: 15479264

11. Easterling DR, Evans JL, Groisman PY, Karl TR, Kunkel KE, Ambenje P. Observed Variability and Trends in Extreme Climate Events: A Brief Review *. Bull. Amer. Meteor. Soc. 2000; 81(3):417–25.

12. D'Amato G, Liccardi G, D'Amato M, Cazzola M. The role of outdoor air pollution and climatic changes on the rising trends in respiratory allergy. Respir Med 2001; 95(7):606–11. https://doi.org/10.1053/rmed.2001.1112 PMID: 11453319

13. Knapp AK, Briggs JM, Koelliker JK. Frequency and Extent of Water Limitation to Primary Production in a Mesic Temperate Grassland. Ecosystems 2001; 4(1):19–28.

14. Menzel A. The allergen riddle. Nat Ecol Evol 2019; 3(5):716–7. https://doi.org/10.1038/s41559-019-0873-7 PMID: 30962559

15. Prank M, Chapman DS, Bullock JM, Belmonte J, Berger U, Dahl A, et al. An operational model for forecasting ragweed pollen release and dispersion in Europe. Agricultural and Forest Meteorology 2013; 182–183:43–53.

16. Minero FJG, Candau P, Tomás C, Morales J. Airborne grass (Poaceae) pollen in southern Spain. Results of a 10-year study (1987?: 96). Allergy 1998; 53(3):266–74. https://doi.org/10.1111/j.1398-9995.1998.tb03886.x PMID: 9542606

17. Gehrig R. The influence of the hot and dry summer 2003 on the pollen season in Switzerland. Aerobiologia 2006; 22(1):27–34.

18. Schwartz MD. Phenology: An Integrative Environmental Science. Dordrecht: Springer Netherlands; 2013.

19. García-Mozo H, Oteros JA, Galán C. Impact of land cover changes and climate on the main airborne pollen types in Southern Spain. Sci Total Environ 2016; 548–549:221–8. https://doi.org/10.1016/j.scitotenv.2016.01.005 PMID: 26802350

20. Matyasovszky I, Makra L, Csépe Z, Sümeghy Z, Deák ÁJ, Pál-Molnár E, et al. Plants remember past weather: A study for atmospheric pollen concentrations of Ambrosia, Poaceae and Populus. Theor Appl Climatol 2015; 122(1–2):181–93.

21. Recio M, Docampo S, García-Sánchez J, Trigo MM, Melgar M, Cabezudo B. Influence of temperature, rainfall and wind trends on grass pollination in Malaga (western Mediterranean coast). Agricultural and Forest Meteorology 2010; 150(7–8):931–40.

22. Makra L, Csépe Z, Matyasovszky I, Deak AJ, Elemér PM, Tusnády G. Interdiurnal variability of Artemisia, Betula and Poaceae pollen counts and their association with meteorological parameters. Carpathian J Earth Environ Sci 2014; 9(3):207–20.

23. Craine JM, Towne EG, Nippert JB. Climate controls on grass culm production over a quarter century in a tallgrass prairie. Ecology 2010; 91(7):2132–40. https://doi.org/10.1890/09-1242.1 PMID: 20715635

24. Alan Ş, Şahin AA, Sarışahin T, Şahin S, Kaplan A, Pınar NM. The effect of geographical and climatic properties on grass pollen and Phl p 5 allergen release. Int J Biometeorol 2018; 62(7):1325–37. https://doi.org/10.1007/s00484-018-1536-0 PMID: 29626255

25. Linares C, Díaz de la Guardia C, Nieto Lugilde D, Alba F. Airborne study of grass allergen (Lol p 1) in different-sized particles. Int Arch Allergy Immunol 2010; 152(1):49–57. https://doi.org/10.1159/000260083 PMID: 19940505

**26.** Rodríguez-Rajo FJ, Jato V, González-Parrado Z, Elvira-Rendueles B, Moreno-Grau S, Vega-Maray A, et al. The combination of airborne pollen and allergen quantification to reliably assess the real pollinosis risk in different bioclimatic areas. Aerobiologia 2011; 27(1):1–12.

**27.** El Kelish A, Zhao F, Heller W, Durner J, Winkler JB, Behrendt H et al. Ragweed (Ambrosia artemisiifolia) pollen allergenicity: SuperSAGE transcriptomic analysis upon elevated CO2 and drought stress. BMC Plant Biol 2014; 14:176. https://doi.org/10.1186/1471-2229-14-176 PMID: 24972689

**28.** Chen M, Xu J, Devis D, Shi J, Ren K, Searle I, et al. Origin and Functional Prediction of Pollen Allergens in Plants. Plant Physiol 2016; 172(1):341–57. https://doi.org/10.1104/pp.16.00625 PMID: 27436829

**29.** Hrabina M, Peltre G, van Ree R, Moingeon P. Grass pollen allergens. Clinical & Experimental Allergy Reviews 2008; 8(1):7–11.

**30.** Duffort O, Quintana J, Ipsen H, Barber D, Polo F. Antigenic similarity among group 1 allergens from grasses and quantitation ELISA using monoclonal antibodies to Phl p 1. Int Arch Allergy Immunol 2008; 145(4):283–90. https://doi.org/10.1159/000110887 PMID: 18004069

**31.** Marcucci F, Sensi L, Incorvaia C, Dell'Albani I, Di Cara G, Frati F. Specific IgE response to different grass pollen allergen components in children undergoing sublingual immunotherapy. Clin Mol Allergy 2012; 10(1):7. https://doi.org/10.1186/1476-7961-10-7 PMID: 22694773

**32.** Petersen A, Schramm G, Bufe A, Schlaak M, Becker W. Structural investigations of the major allergen I on the complementary DNA and protein level. Journal of Allergy and Clinical Immunology 1995; 95 (5):987–94. https://doi.org/10.1016/s0091-6749(95)70099-4 PMID: 7751520

**33.** Taeger S, Sparks TH, Menzel A. Effects of temperature and drought manipulations on seedlings of Scots pine provenances. Plant Biol (Stuttg) 2015; 17(2):361–72.

**34.** Martínez-Sancho E, Vásconez Navas LK, Seidel H, Dorado-Liñán I, Menzel A. Responses of Contrasting Tree Functional Types to Air Warming and Drought. Forests 2017; 8(11):450.

**35.** Meier U. Entwicklungsstadien mono- und dikotyler Pflanzen [Die erweiterte BBCH Monographie 2]; 2001.

**36.** Schindelin J, Arganda-Carreras I, Frise E, Kaynig V, Longair M, Pietzsch T et al. Fiji: an open-source platform for biological-image analysis. Nat Methods 2012; 9(7):676–82. https://doi.org/10.1038/nmeth.2019 PMID: 22743772

**37.** Rueden CT, Schindelin J, Hiner MC, DeZonia BE, Walter AE, Arena ET et al. ImageJ2: ImageJ for the next generation of scientific image data. BMC Bioinformatics 2017; 18(1):529. https://doi.org/10.1186/s12859-017-1934-z PMID: 29187165

**38.** Jung S, Estrella N, Pfaffl MW, Hartmann S, Handelshauser E, Menzel A. Grass pollen production and group V allergen content of agriculturally relevant species and cultivars. PLoS One 2018; 13(3): e0193958. https://doi.org/10.1371/journal.pone.0193958 PMID: 29529096

**39.** Albertine JM, Manning WJ, DaCosta M, Stinson KA, Muilenberg ML, Rogers CA. Projected carbon dioxide to increase grass pollen and allergen exposure despite higher ozone levels. PLoS One 2014; 9(11): e111712. https://doi.org/10.1371/journal.pone.0111712 PMID: 25372614

**40.** Fahlbusch B, Hornung D, Heinrich J, Dahse HM, Jäger L. Quantification of group 5 grass pollen allergens in house dust. Clin Exp Allergy 2000; 30(11):1646–52. https://doi.org/10.1046/j.1365-2222.2000.00926.x PMID: 11069575

**41.** Chapman MD. Allergen specific monoclonal antibodies: New tools for the management of allergic disease. Allergy 1988; 43(Suppl. 1):7–14. https://doi.org/10.1111/j.1398-9995.1988.tb05042.x PMID: 3354797

**42.** Schäppi GF, Taylor PE, Pain MC, Cameron PA, Dent AW, Staff IA et al. Concentrations of major grass group 5 allergens in pollen grains and atmospheric particles: implications for hay fever and allergic asthma sufferers sensitized to grass pollen allergens. Clin Exp Allergy 1999; 29(5):633–41. https://doi.org/10.1046/j.1365-2222.1999.00567.x PMID: 10231323

**43.** Fahlbusch B, Müller WD, Schlenvoigt G, Jäger L, Wahl R, Weber B. Monoclonal antibody immunoassay for quantitative analysis of group V allergens in grass pollen extracts. Clin Exp Allergy 1993; 23(9):747–54. https://doi.org/10.1111/j.1365-2222.1993.tb00362.x PMID: 10779305

**44.** Schäppi GF, Monn C, Wuthrich B, Wanner HU. Direct determination of allergens in ambient aerosols: methodological aspects. Int Arch Allergy Immunol 1996; 110(4):364–70. https://doi.org/10.1159/000237329 PMID: 8768804

**45.** Flicker S, Vrtala S, Steinberger P, Vangelista L, Bufe A, Petersen A, et al. A human monoclonal IgE antibody defines a highly allergenic fragment of the major timothy grass pollen allergen, Phl p 5: molecular, immunological, and structural characterization of the epitope-containing domain. J Immunol 2000; 165 (7):3849–59. https://doi.org/10.4049/jimmunol.165.7.3849 PMID: 11034391

**46.** Fahlbusch B, Schlenvoigt G, Müller WD, Weber B, Jäger L. A two-site monoclonal antibody ELISA for the quantification of group V allergens in grass extracts. Clin Exp Allergy 1994; 24(8):752–7. https://doi.org/10.1111/j.1365-2222.1994.tb00986.x PMID: 7982125

47. Klysner S, Welinder KG, Lowenstein H, Matthiesen F. Group V allergens in grass pollens: IV. Similarities in amino acid compositions and NH2-terminal sequences of the group V allergens from Lolium perenne, Poa pratensis and Dactylis glomerata. Clin Exp Allergy 1992; 22(4):491–7. https://doi.org/10.1111/j.1365-2222.1992.tb00152.x PMID: 1611548

48. R Core Team. R: A Language and Environment for Statistical Computing. Vienna, Austria; 2015. URL: https://www.R-project.org/.

49. Aloisi I, Del Duca S, Nuntiis P, Mandrioli P, Fernández-González D. Comparison of extraction methods for Poaceae pollen allergens. Aerobiologia 2018; 178(1):231.

50. Menzel A, Yuan Y, Matiu M, Sparks T, Scheifinger H, Gehrig R, et al. Climate change fingerprints in recent European plant phenology. Glob Chang Biol 2020. https://doi.org/10.1111/gcb.15000 PMID: 31950538

51. Cui T, Martz L, Guo X. Grassland Phenology Response to Drought in the Canadian Prairies. Remote Sensing 2017; 9(12):1258.

52. Bundessortenamt, Osterfelddamm 80, 30627 Hannover. Beschreibende Sortenliste „Rasengräser 2019“; 2019.

53. Bundessortenamt, Osterfelddamm 80, 30627 Hannover. Beschreibende Sortenliste “Futtergräser Esparsette, Klee, Luzerne 2018”; 2018.
